# *In vitro* antifungal susceptibility of yeasts and molds isolated from sputum of tuberculosis relapse and retreatment patients

**DOI:** 10.11604/pamj.2021.38.227.26485

**Published:** 2021-03-01

**Authors:** Josephat Tonui, Marianne Mureithi, Walter Jaoko, Christine Bii

**Affiliations:** 1 University of Nairobi, School of Medicine, Department of Medical Microbiology, Nairobi, Kenya,; 2 Kenya Medical Research Institute, Centre for Microbiology Research, Mycology Unit, Nairobi, Kenya

**Keywords:** Yeast, molds, antifungals, susceptibility, tuberculosis, retreatment

## Abstract

**Introduction:**

opportunistic fungal infections due to immunosuppression coupled with antifungal drug resistance are an emerging challenge globally. The present study examined the antifungal susceptibility of yeasts and molds from sputum of tuberculosis retreatment and relapse patients at selected reference facilities in Kenya.

**Methods:**

a total of 340 sputa samples from patients who gave written informed consent were examined. Fungal culture was done on sabouraud dextrose agar (SDA). Molds were identified by macroscopic and microscopic features while yeasts were inoculated on CHROM^TM^agar Candida and confirmed using API 20C AUX^TM^. Itraconazole (ICZ), voriconazole (VCZ), fluconazole (FCZ) and amphotericin B (AMB) were tested using broth micro-dilution methods according to Clinical and Laboratory Standards Institute (CLSI).

**Results:**

out of the 340 samples, 14.4% (n=49) and 15.6% (n=53) were positive for yeasts and molds respectively. Candida albicans and C. krusei were the most predominant isolates constituting 49.0% (n=24) and 20.4% (n=10) of the total yeasts respectively. Aspergillus spp. were the most frequent (22.6%) molds and isolates with MICs ≥4μg/ml on the antifungal agents were noted. All the molds except two (n=2) isolates of Scedosporium aspiopermum exhibited MICs >4μg/ml for fluconazole. Overall, molds were more sensitive to AMB and VCZ. Candida albicans had MIC_50_ <0.06μg/ml, and MIC_90_<4μg/ml. There was a statistically significant difference (F=3.7, P=0.004<0.05) in the overall sensitivity pattern of molds for the four antifungal agents while there was no significant difference (F=1.7, P=0.154>0.05) in sensitivity exhibited by the yeasts.

**Conclusion:**

the study demonstrates the significance of fungal colonization in presumptive tuberculosis retreatment or relapse with evidence of triazole resistance. There is need to strengthen fungal diagnostic and clinical management capabilities in susceptible populations.

## Introduction

Fungal diseases contributes to significant morbidity and mortality in humans and animals [1]. Recently, cases of superficial and systemic fungal infections have escalated with a possibility of reaching epidemic magnitude [2]. The most affected members of society are the immunocompromised and those undergoing invasive medical procedures as well as infants [3]. Current knowledge depicts an increase in pulmonary mycoses as a co-morbidity or a sequelae of TB that frequently leads to mortality [4]. Lung infection with fungi mainly occurs following inhalation of airborne fungal propagules that are ubiquitous in the environment [5]. Numerous yeasts and molds have been implicated in pulmonary pathologies and the clinical features and severity of disease varies from asymptomatic in otherwise healthy individuals to overt symptoms such as cough, dyspnea, chest pain, and bronchiectasis [6]. These symptoms exhibited are usually non-specific and are also consistent with other pulmonary conditions including TB. Some of the significant fungi of clinical importance in pulmonary conditions includes; *Aspergillus spp (A. fumigatus A. flavus and A. niger)*, yeasts mainly *Cryptococcus spp. (C. neoformans)* and *Candida spp (C. albicans and C. tropicalis)*. Other significant pathogens include *Mucor spp* and *Histoplasma capsulatum* [7].

Fungal pathogens have evolved substantially over the years exhibiting greater and increased resistance to the few existing antifungal agents [8]. The trend is worrisome to clinicians and patients as well as microbiologists and other researchers alike in an effort to understand this phenomenon [9]. The big challenge in management of pulmonary fungal infections is emerging triazole resistance especially among the *Aspergillus fumigatus* [1, 10]. This is worsened in developing countries by resource constraints, scarcity of quality medicine and poor health care systems [11]. There are different classes of antifungal medications with varying clinical spectrum and mode of action. Four main categories of antifungals are currently in clinical use namely azoles, allylamines, polyenes and echinocandins. Additionally, there exists a diverse group of other compounds, such as flucytosine and griseofulvin [12]. The development of new antifungal regimen has been hampered by several factors particularly limited selective toxicity of candidate compounds as well as primary and secondary resistance [13]. Fungi are eukaryotic organisms sharing substantial structural features with mammalian cells hence making it difficult to target the fungal cells without significant harm to the host.

In contrast, prokaryotes mainly bacteria offer a wide variety of metabolic and structural components that can be targeted selectively thus a wide range of antibiotics are available [14]. Antifungal drugs exert their activity on various biosynthetic processes within the cell or interfere with ergosterol a vital component of the fungal cell membrane leading to loss of integrity and consequential death. Mechanisms of resistance that have been described encompass alteration of drug targets, altered drug uptake or efflux, and lack of microbial enzymes to metabolize the drug to an active form [15]. Screening methods to determine the *in vitro* and *in vivo* efficacy of antifungals are still undergoing standardization and micro-dilution techniques are the gold standard. However significant progress has been achieved so far for specific fungal pathogens such as *Candida spp*. and drugs [8]. It is essential to understand the sensitivity of a fungal etiological agent implicated in an infectious process for selection of appropriate treatment. In view of the impending antifungal resistance, the current study reports on resistance among yeasts and molds from sputum of tuberculosis retreatment and relapse cases to three azole antifungals; fluconazole (FCZ), voriconazole (VCZ) and itraconazole (ICZ), and the polyene-amphotericin B (AMB).

## Methods

**Study samples:** the current study was cross-sectional and laboratory investigations were carried out at Kenya Medical Research Institute (KEMRI), Center for Microbiology Research-Mycology Unit. Expectorated sputum samples were obtained from tuberculosis retreatment and relapse cases who presented with clinical symptoms typical of pulmonary tuberculosis and with a history of tuberculosis treatment. The participants were recruited from three reference facilities in Kenya namely Moi Teaching and Referral Hospital, Mbagathi Hospital and the National TB Reference Laboratory and all were above 18 years of age. The samples were transported promptly to the mycology laboratory according to the standard operating procedures for collection, biosafety and shipment of infectious material. The study was reviewed and approved by Kenyatta National Hospital/University of Nairobi Ethics Review Committee (KNH/UoN-ERC) (study number P108/02/2018). Written consent was obtained for all patients and personal information was handled with utmost confidentiality.

**Isolation and identification of fungi:** sputum samples were digested with sputasol and subsequently inoculated in duplicate on sabouraud dextrose agar (SDA) supplemented with 0.65 ml gentamicin (40 mg/ml) and 10 ml of chloramphenicol. The cultures were incubated at 30°C and 35°C and monitored for fungal growth for up to four weeks. Mold isolates were identified by morphological colony growth characteristics such as topography, color on the reverse and obverse as well as microscopic features on lactophenol cotton blue mount [16]. Yeasts were sub cultured on CHROMagar^TM^ Candida for presumptive identification by morphologic characteristics such as color, size of colonies and shape of the cells. India Ink was performed to differentiate *Cryptococcus neoformans* based on the presence of a capsule as per the available standard operating procedures. Confirmation of the identity of yeasts was carried out using Analytical Profile Index (API 20 C AUX-bioMerieux Durham, USA).

**Antifungal susceptibility testing:** antifungal susceptibility was performed using broth micro-dilution techniques according to Clinical and Laboratory Standards Institute guidelines (CLSI), M27-A3 and M38-A2 respectively [17, 18]. The antifungal drugs tested were; voriconazole, itraconazole, fluconazole and amphotericin B. voriconazole, itraconazole and amphotericin B were diluted in analytical grade dimethyl sulfoxide (DMSO) while fluconazole was dissolved in sterile distilled water. The dilutions were prepared in the range of 0.03 to 32 µg/ml. Sterile test tubes were used for drug dilutions and sterile disposable multi-well microdilution plates (96 U-shaped wells) were utilized for susceptibility assays. The final required drug concentration was achieved with the RPMI media. Drugs were diluted in tryptic soy broth (TSB) for mold testing which gave distinct endpoints and RPMI 1640 for yeasts. 200μl of broth containing the drug were dispensed in the test wells and drug free media was dispensed in the positive and negative control wells. A 0.5 McFarland of inoculums were prepared from pure cultures of the test fungi and 10 μl were inoculated from the lowest to highest drug concentration on the micro-titer wells. The micro-titer wells were incubated at 35°C for yeast testing and 30°C for molds and thereafter the MICs were read at 48 hours. *Aspergillus flavus* ATCC^®^204304 and *Candida parapsilosis*-ATCC^®^22019 strains were included for quality control based on their defined minimum inhibitory concentrations (MICs). The end point was determined as the lowest concentration that prevented visible growth for amphotericin B, voriconazole, and itraconazole while the MIC for fluconazole was determined as the lowest concentration corresponding to 50% reduction in turbidity compared to the control well.

## Results

Out of the 340 sputa samples tested, 14.4% (n=49-Table 1) and 15.6% (n=53-Table 2) were positive for yeasts and molds respectively. *Candida albicans* and *C. krusei* were the most predominant yeast species isolated constituting 48.9% (n=24) and 20.4% (n=10) of the total yeast isolates respectively (Table 2). On the other hand, *Aspergillus flavus* and *A. niger* were the most frequent mold species recovered making up 22.6% (n=12) and 15.0% (n=8) respectively (Table 1). Some molds exhibited MICs ≥4μg/ml that were considered resistant to azoles (Table 1). For amphotericin B (AMB), 41.6% (n=5) of *A. flavus* had MICs ≥4μg/ml, while 12.5% (n=1) of *A. niger*, 100% (n=3) of *A. versicolor* and 40% (n=2) of *Penicilium spp*. showed similar MIC results for AMB (Table 1).

**Table 1:** distribution of minimum inhibitory concentrations (MICs) of filamentous fungal isolates

Antifungal agent and isolate	No. of isolates with respective MICs (μg/ml) n=53 (48 hours reading)												
**Amphotericin B**	**<0.06**	**0.12**	**0.25**	**0.5**	**1**	**2**	**4**	**8**	**16**	**>32**	**Total**	**MIC_50_**	**MIC_90_**
*Aspergillus flavus*	1	-	2	-	2	2	1	1	2	1	12	1	8
*Aspergillus niger*	1	-	0	-	2	4	1	-	-		8	2	2
*Aspergillus vesicolor*	-	-	-	-	-	-		1	-	2	3	8	>32
*Aspergillus terreus*	-	-	-	-	-	-	1	1	1	2	5	16	>32
*Aspergillus candidus*	-	-	-	-	-	-	1	1	-	-	2	4	8
*Aspergillus fumigatus*	1	1	1	-	-	1	1	-	-	-	5	0.25	2
*Mucor racemosus*	2	-	-	-	-	2	-	-	-	-	4	2	2
*Paecilomyces variotii*	-	-	1	3	1	-	-	-	-	-	5	0.5	0.5
*Scedosporium aspeospermum*	3	-	-	-	-	-	-	-	-	-	3	<0.06	<0.06
*Penicillium spp*	1	-	1	1	0	-	-	-	1	1	5	0.5	16
*Aspergillus flavus* (ATCC® 204304)	**-**	**-**	**-**	-	1	**-**	**-**	**-**	**-**	**-**	1	1	1
**Itraconazole**	**<0.06**	**0.12**	**0.25**	**0.5**	**1**	**2**	**4**	**8**	**16**	**>32**	**Total**	**MIC_50_**	**MIC_90_**
*Aspergillus flavus*	3	2	-	1	3	-	2	-	1	-	12	0.5	1
*Aspergillus niger*	1	1	1	1	2	2	-	-	-	-	8	0.5	2
*Aspergillus vesicolor*	-	-	-	-	2	-	-	1	-	-	3	1	8
*Aspergillus terreus*	-	-	-	2	3	-	-	-	-	-	5	0.5	1
*Aspergillus candidus*	1	-	-	-	-	-	-	1	-	-	2	<0.06	8
*Aspergillus fumigatus*	1	-	-	1	1	1	1	-	-	-	5	1	2
*Mucor racemosus*	-	-	-	2	-	2	-	-	-	-	4	0.5	2
*Paecilomyces variotii*	4	-	-	-	1	-	-	-	-	-	5	<0.06	<0.06
*Scedosporium aspeospermum*	3	-	-	-	-	-	-	-	-	-	3	<0.06	<0.06
*Penicillium spp*	-	1	-	-	1	2	1	-	-	-	5	2	2
*Aspergillus flavus* (ATCC® 204304)	**-**	**-**	1	**-**	**-**	**-**	**-**	**-**	**-**	**-**	1	0.25	0.25
**Voriconazole**	**<0.06**	**0.12**	**0.25**	**0.5**	**1**	**2**	**4**	**8**	**16**	**>32**	**Total**	**MIC_50_**	**MIC_90_**
*Aspergillus flavus*	4	2	-	-	2	4	-	-	-	-	12	<0.12	2
*Aspergillus niger*	-	-	-	2	5	1	-	-	-	-	8	1	1
*Aspergillus vesicolor*	-	-	-	-	2	1	-	-	-	-	3	1	2
*Aspergillus terreus*	-	-	-	-	2	2	1	-	-	-	5	2	2
*Aspergillus candidus*	-	-	-	1	-	1	-	-	-	-	2	0.5	2
*Aspergillus fumigatus*	1	-	1	1	2	-	-	-	-	-	5	0.5	1
*Mucor racemosus*	-	-	-	-	-	1	-	1	2	-	4	8	16
*Paecilomyces variotii*	1	-	-	1	-	1	1	-	-	1	5	2	4
*Scedosporium aspeospermum*	3	-	-	-	-	-	-	-	-	-	3	<0.06	<0.06
*Penicillium spp*.	-	-	1	-	1	-	1	1	-	1	5	4	8
*Aspergillus flavus* (ATCC® 204304)	**-**	**-**	**-**	**-**	**-**	1	-	-	-	-	1	2	2
**Fluconazole**	**<0.06**	**0.12**	**0.25**	**0.5**	**1**	**2**	**4**	**8**	**16**	**>32**	**Total**	**MIC_50_**	**MIC_90_**
*Aspergillus flavus*	-		-	-	-	-		2	1	9	12	>32	>32
*Aspergillus niger*	-	-	-	-	-	-	-	-	1	7	8	>32	>32
*Aspergillus vesicolor*	-	-	-	-	-	-	-	-	-	3	3	>32	>32
*Aspergillus terreus*	-	-	-	-	-	-	-	1	-	4	5	>32	>32
*Aspergillus candidus*	-	-	-	-	-	-	-	-	-	2	2	>32	>32
*Aspergillus fumigatus*	-	-	-	-	-	-	-	-	-	5	5	>32	>32
*Mucor racemosus*	-	-	-	-	-	-	-	-	-	4	4	>32	>32
*Paecilomyces variotii*	-	-	-	-	-	-	-	-	-	5	5	>32	>32
*Scedosporium aspeospermum*	-	-	-	2	-	-	-	1	-	-	3	0.5	8
*Penicillium spp*	-	-	-	-	-	-	-	-	-	5	5	>32	>32
*Aspergillus flavus* (ATCC®204304)	**-**	**-**	**-**	**-**	**-**	**-**	**-**	**-**	1	-	1	16	16

Quality control strain *Aspergillus flavus* ATCC**®**204304 MIC range AMB (0.5-4), ICZ (0.25-0.5), VCZ (0.5-4), MIC_50_-drug concentration that inhibited 50% of isolates, MIC_90_- drug concentration that inhibited 90% of isolates.

**Table 2:** minimum inhibitory concentrations (MICs) of yeast isolates on the antifungal agents tested

Antifungal agent and yeast species	Number of isolates with respective MICs (μg/ml) n=49 (48 hours reading)												
**Amphotericin B**	**<0.06**	**0.12**	**0.25**	**0.5**	**1**	**2**	**4**	**8**	**16**	**>32**	**Total**	**MIC_50_**	**MIC_90_**
*Candida albicans*	13	-	2	1	-	3	4	1	-	-	24	<0.06	4
*Candida krusei*	2	1	1	1	-	2	1	1	-	1	10	0.5	8
*Candida tropicalis*	2	-	-	2	2	1	-	-	-	-	7	0.5	2
*Candida famata*	-	-	-	-	-	-	2	-	-	-	2	4	4
*Candida zeylanoids*	1	-	-	1	-	-	-	-	-	-	2	<0.06	0.5
*Candida lusitaniae*	-	-	-	-	-	1	-	-	-	-	1	2	2
*Cryptococcus neoformans*	1	-	-	-	-	-	-	-	-	-	1	<0.06	<0.06
*Candida guilliermondii*	-	-	-	-	-	1	-	-	-	-	1	2	2
*Candida parapsilosis*	-	-	-	-	-	-	1	-	-	-	1	4	4
*Candida parapsilosi*s-ATCC®22019	**-**	**-**	**-**	**-**	**-**	1	**-**	**-**	**-**	**-**	1	2	2
**Itraconazole**	**<0.06**	**0.12**	**0.25**	**0.5**	**1**	**2**	**4**	**8**	**16**	**>32**	**Total**	**MIC_50_**	**MIC_90_**
*Candida albicans*	22S	-	-	-	-	1R	-	-	-	1R	24	<0.06	<0.06
*Candida krusei*	4S	2S	-	-	2R	-	-	-	-	2R	10	0.12	>32
*Candida tropicalis*	6S	-	1S	-	-	-	-	-	-	-	7	<0.06	<0.06
*Candida famata*	2	-	-	-	-	-	-	-	-	-	2	<0.06	<0.06
*Candida zeylanoids*	1	-	1	-	-	-	-	-	-	-	2	<0.06	0.25
*Candida lusitaniae*	1	-	-	-	-	-	-	-	-	-	1	<0.06	<0.06
*Cryptococcus neoformans*	1	-	-	-	-	-	-	-	-	-	1	<0.06	<0.06
*Candida guilliermondii*	-	-	-	1	-	-	-	-	-	-	1	0.5	0.5
*Candida parapsilosis*	1S	-	-	-	-	-	-	-	-	-	1	<0.06	<0.06
*Candida parapsilosis*-ATCC®22019	**-**	**-**	1S	**-**	**-**	**-**	**-**	**-**	**-**	-	1	0.25	0.25
**Voriconazole**	**<0.06**	**0.12**	**0.25**	**0.5**	**1**	**2**	**4**	**8**	**16**	**>32**	**Total**	**MIC_50_**	**MIC_90_**
*Candida albicans*	15S	-	2S	4S	1R	-	1R	-	-	1R	24	<0.06	0.5
*Candida krusei*	2S	-	3S	1S	-	-	-	1R	-	3R	10	0.25	>32
*Candida tropicalis*	4S	-	-	1R	1R	-	-	-	-	1R	7	<0.06	-
*Candida famata*	-	-	-	2	-	-	-	-	-	-	2	0.5	0.5
*Candida zeylanoides*	1	-	-	-	1	-	-	-	-	-	2	<0.06	1
*Candida lusitaniae*	1	-	-	-	-	-	-	-	-	-	1	<0.06	<0.06
*Cryptococcus neoformans*	-	-	1	-	-	-	-	-	-	-	1	0.25	<0.25
*Candida guilliermondii*	-	-	1	-	-	-	-	-	-	-	1	0.25	0.25
*Candida parapsilosis*	-	-	-	1R	-	-	-	-	-	-	1	0.5	0.5
*Candida parapsilosis*-ATCC®22019	**-**	1S	**-**	**-**	**-**	**-**	**-**	**-**	**-**	-	1	0.12	0.12
**Fluconazole**	**<0.06**	**0.12**	**0.25**	**0.5**	**1**	**2**	**4**	**8**	**16**	**>32**	**Total**	**MIC_50_**	**MIC_90_**
*Candida albicans*	22S	2S	-	-	-	-	-	-	-	-	24	<0.06	0.12
*Candida krusei*	3S	-	1S	-	1R	1R	-	-	2R	2R	10	1	>32
*Candida tropicalis*	3S	-	-	2S	1S	-	-	-	-	1R	7	0.5	1
*Candida famata*	-	-	-	-	1	1	-	-	-	-	2	1	2
*Candida zeylanoids*	1	-	-	-	1	-	-	-	-	-	2	<0.06	1
*Candida lusitaniae*	1	-	-	-	-	-	-	-	-	-	1	<0.06	<0.06
*Cryptococcus neoformans*	1	-	-	-	-	-	-	-	-	-	1	<0.06	<0.06
*Candida guilliermondii*	-	1	-	-	-	-	-	-	-	-	1	0.12	0.12
*Candida parapsilosis*	1S	-	-	-	-	-	-	-	-	-	1	<0.06	<0.06
*Candida parapsilosis*-ATCC®22019	**-**	**-**	**-**	1S	-	**-**	**-**	**-**	**-**	**-**	1	0.5	0.5

Quality control (QC) strain *Candida parapsilosis*-ATCC**®**22019 MIC range AMB (0.5-4), ICZ (0.12-0.5), VCZ (0.03-0.25), FCZ (1.0-4.0), R-resistant, S-susceptible, I-Susceptible doze dependent, MIC_50_-drug concentration that inhibited 50% of isolates, MIC_90_- drug concentration that inhibited 90% of isolates.

Equally, mold isolates exhibiting high MICs for itraconazole (ICZ) were identified and *A. flavus* (25%, n=3), *A. versicolor* (33.3%, n=1), *A. candidus* (50%, n=1), and *Penicillium spp*. (20%, n=1) had MIC ≥4μg/ml on ICZ. Similarly, isolates with high MICs to voriconazole (VCZ) were noted mainly *A. terreus* (20%, n=1), *Mucor racemosus* (75%, n=3), *Paecillomyces variotii* (40%, n=2) and *Penicilllium spp*. (60%, n=3). All the molds except two (n=2) isolates of *Scedosporium aspiopermum* exhibited MICs >4μg/ml on fluconazole (Table 1). Yeast isolates showed relatively low MICs to the antifungal agents (Table 2). Twenty-one percent (21%, n=5) of *Candida albicans* had MICs ≥4μg/ml for amphotericin B, 4% (n=1) for itraconazole, 8% (n=2) for voriconazole and none of the *C. albicans* isolates had ≥4μg/ml for fluconazole (Table 2). MICs ≥4μg/ml was also noted for 40% (n=4) of *C. krusei* isolates on both voriconazole and fluconazole, 30% (n=3) on amphotericin B and 20% (n=2) on itraconazole. One isolate of *C. tropicalis* had MIC >32μg/ml on both fluconazole and voriconazole.

**Figure 1:**
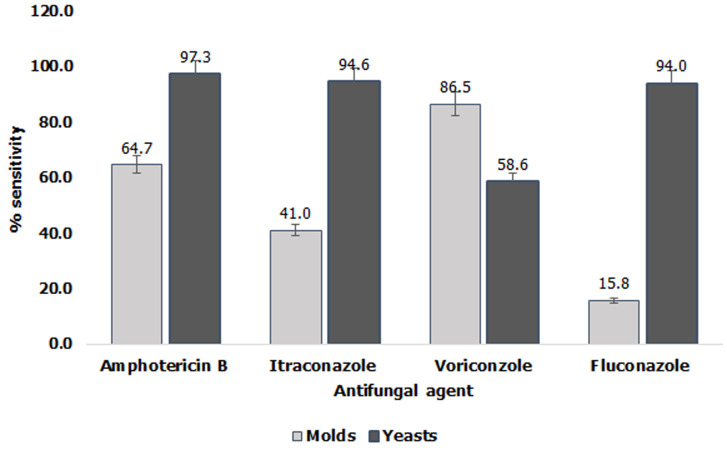
comparative sensitivity of mold and yeast isolates from sputum on four antifungal agents

Other *Candida* isolates with MICs ≥4μg/ml for AMB were *C. famata* (n=2) and *C. parapsilosis* (n=1) (Table 2). The MIC_50_ and MIC_90_ for the molds and the yeast are indicated in the respective tables. Most of the molds had MIC_50_ >32μg/ml for fluconazole while *Candida albicans* exhibited the greatest sensitivity to all the four antifungal agents with MIC *50*, and MIC *90* of <0.06μg/ml and <4μg/ml respectively. Overall, the molds showed greater sensitivity to voriconazole and amphotericin B while the yeasts were more uniformly sensitive (Figure 1). There was a statistically significant difference (F=3.7, P=0.004<0.05) in the sensitivity pattern of the molds to the four antifungal agents while for the yeasts, there was no statistically significant difference (F=1.7, P=0.154>0.05).

## Discussion

Fungal pathogens are increasingly gaining attention due to the rising incidence of fungal diseases and emerging antifungal resistance. The current study reports the antifungal susceptibility patterns of yeast and molds isolated from sputum of tuberculosis relapse and retreatment patients. Pulmonary fungal infections have emerged as significant etiologic agents in the immunocompromised population [1]. The greatest threat to the management of tuberculosis is the occurrence of multi-drug resistance resulting in treatment failure and increase in morbidity and mortality [19]. This scenario has now transitioned to a complex medical dilemma due to the increased recognition of the role of fungal etiologies in pulmonary disease that could be missed or misdiagnosed as tuberculosis based on the clinical picture [20]. Clinicians encounter presumptive pulmonary tuberculosis cases with inadequate bacteriological confirmation and others whose clinical symptoms persist despite compliance with prescribed anti-tuberculosis regimen [21]. In the current study, sputum samples of 340 patients who had a history of tuberculosis treatment yielded diverse yeasts n=49 (14.4%) and molds n=53 (15.6%). This represents a significant airway colonization, sensitization and possible invasive infection by these fungi. Fungal spores are ubiquitous in the environment and exposure occurs mainly through inhalation while yeasts such as *Candida albicans* is part of the human a normal flora. There is growing evidence based on non-culture sequencing techniques that the lung mycobiome contributes immensely to the impact of chronic respiratory diseases [22]. The outcome of fungal lung colonization depends on the interplay with the immune system and other existing pulmonary diseases including tuberculosis [23-25]. Fungal pathogens such as; *Aspergillus spp, Penicilllium spp, Mucor racemosus, Paecillomyces variotii* and *Scedosporium aspiopermum* were recovered from sputum. Isolation of yeasts and molds from high-risk patients has been linked with invasive pathology. *Scedosporium aspeopermum* and zygomycete infections though rare do occur and are associated with high mortality rate of greater than 60% [26].

In the present study, four antifungal agents namely amphotericin B, itraconazole, voriconazole and fluconazole were tested against fungal isolates from sputum. Treatment of fungal infections with an antifungal agent depends on the clinical condition of a patient and may also be used as prophylaxis in high risk conditions [12]. Similar to antibacterial resistance, antifungal resistance is an emerging challenge complicating the antimicrobial resistance control agenda [27]. Emerging triazole resistance in *Aspergillus fumigatus* has been noted ascribed to fungicide use in agriculture [28, 29]. The current study identified respiratory fungal isolates exhibiting resistance and high MICs to the antifungal agents. *Aspergillus flavus* (41.6%), and *Candida albicans* (21%) exhibited MICs ≥4μg/ml to *Amphotericin B while all the A. versicolor* isolates were resistant to Amphotericin B (Table 1). All the filamentous fungi isolated except *Paecillomyces variotii* and *Penicillium spp*. were susceptible to triazoles (voriconazole and itraconazole) suggesting favorable therapeutic options for management of mold infections. The two were resistant to triazoles and exhibited MIC >32 μg/ml (Table 1). Triazole resistance especially in *A. fumigatus* is an emerging challenge in clinical practice [30]. Cultures that turn positive for *Aspergilli* during the course of treatment with voriconazole have been associated with treatment failure, resistance and subsequent mortality [31].

Based on the CLSI interpretive criteria for the most predominant yeast isolates in the current study, 92% (n=22), 71% (n=21), and 100% (n=24) of *Candida albicans* were susceptible to itraconazole, voriconazole and fluconazole respectively. Only 60% of *C. krusei* were susceptible to both itraconazole and voriconazole with a decreased susceptibility to fluconazole (40%) (Table 2) [17]. Several studies have reported fluconazole resistance among *C. albicans* ascribed to prophylactic use of fluconazole in HIV patients [32]. There is evidence of fluconazole resistance among clinical *Candida* species in Kenya and Tanzania [33, 34]. All *C. tropicalis* isolates were susceptible to itraconazole (100%, n=7) and 57% (n=4) to voriconazole. It is good to note that the cost of triazole antifungals is prohibitive and their availability is limited, hence this could explain the high sensitivity observed in this study. Nevertheless, increasing azole resistance is being encountered clinically ascribed to prophylactic use of azoles in immunocompromised patients and use of azole-based fungicides in agriculture. The need for routine and constant antifungal resistance testing is becoming essential to predict the prognosis of fungal infections and the outcome of treatment choices.

*In vitro* antifungal sensitivity testing is meant to facilitate the correlation of in vivo efficacy of therapy, track the occurrence of resistance in fungal etiological agents and to assess the therapeutic potential of new molecules [35]. A major consideration in antifungal susceptibility testing is the clinical relevance of *in vitro* MICs. The correlation of increased MICs with molecular resistance to triazole antifungals and treatment failure has been recognized particularly for *Aspergillus spp*. [36]. Itraconazole is mainly used for treatment of chronic pulmonary aspergilllosis and voriconazole has been used as first-line regimen for invasive aspergillosis (IA). New molecules introduced in the recent years include isavuconazole, accepted for treatment of IA and posaconazole administered as a prophylaxis in patients with increased risk of infection such as acute myeloid leukemia [28]. The challenge with newer triazole in resource constrained setting is the cost and availability to those who need it most. Therefore, resistance to triazoles is unlikely to arise due to clinical use but due to irrational use of triazole based fungicides in agriculture.

Antifungal susceptibility testing (AST) using broth microdilution techniques as reference standards by the Clinical Laboratory Standards Institute (CLSI) and European Committee on Antibiotic Susceptibility Testing (EUCAST) is a challenge in our setting. First, the procedures for performing AST with microdilution are laborious and time consuming hence the turn around time for results is signficantly dawdling. Secondly, other impediments include the unpredictable correlation between treatment outcome and *in vitro* MICs results. The limited capabilities for diagnosis, treatment and monitoring of antifungal treatment and resistance is still limited in Kenya requiring investment in technical capabilities in fungal diagnosis and management. With the advent of antimicrobial resistance (AMR), it has been agued that it would be imposible to adress the key challlenges and alleviate the increasing menace without sufficient attention to diagnosis and management of fungal diseases [37]. Inacurate diagnosis of fungal sepsis in hospital settings and administration of broad spectrum antibiotics, inability to diagnose chronic pulmonary aspergillosis in presumptive tuberculosis patients and subsequent administration of anti-tubercular drugs and misdiagnosis of fungal related asthma constitute the vital drivers of AMR [37].

Antifungal resistance varies significantly, depending on the fungal agent and the geographical region, a great variability in this phenomenon has been observed in *Candida glabrata* following the introduction of new antifungals particularly echinocandins [15]. This species was however not isolated in the current study. *Candida auris*, a newly emerging pathogen exhibits signficantly high resistance to a wide range of antifungal agents and is highly transmisible aphenomenon often seen in pathogenic bacteria [38]. One key strategy to adress the problem of antifungal resistance is the establishment of antifungal steewardship in clinical settings to keep track and reduce the overall use of antiffungal drugs, as well as adoption of proper infection prevetion and control strategies [39]. Successfully implemnted antifungal stewardship programmes have been acompanied by substancial benefits in minimizing healthcare costs, improving patient outcomes and mitigation of toxicities resulting from unnecessary antifungal prescriptions [40]. Considerable emphasis has been placed on antibiotic resitance disregarding antifungal stewardship. Antifungal drugs are relatively expensive medicines and a well implimented stewardship program would be beneficial in all facets [41]. Resource contrains and lack of adequate incentives for clinical personnel to carry out satisfactory antimicrobial stewardship activities are among the greatest challenges to successful implementation of such programs [42]. Further, the role of the increased use of agricultural fungicides in the emergence of clinical antifungal resistance should be adressed adequately as there is evidence to suggest azoles used in agriculture as the main drivers [14, 43-45].

## Conclusion

The present study underscores the need to address the emerging clinical challenge of fungal pulmonary infections and antifungal resistance. These infections are often missed or misdiagnosed as tuberculosis and disregarded as contaminants in tuberculosis culture laboratories. Fungi exhibiting high MICs to the antifungals tested, represents an imminent threat to antifungal therapy hence resistance testing is vital for the selection of appropriate antifungal treatment. However, correlation of microbiological resistance and clinical outcome as well as pulmonary fungal colonization of retreatment and relapse patients is essential. Overall, a greater sensitivity to amphotericin B and voriconazole was observed in molds while the yeasts, mainly *C. albicans*, showed nearly uniform sensitivity to the four drugs tested. Further studies should be carried out to determine aspects such as the molecular basis of the resistance as well as the responsible genes. Some species of fungi are intrinsically resistant to certain antifungals while some acquire resistance due to irrational use. Therefore, it is crucial to establish functional antimicrobial stewardship programs focusing on rational use of both antifungals and antibiotics to avert the emergence of resistant strains and to reduce associated patient and healthcare costs.

### What is known about this topic

- Antifungal resistance is now an emerging problem and resistance has been documented from both clinical and environmental fungal isolates particularly to azole antifungal agents;
- The prevalence of opportunistic fungal infections has increased markedly in the recent years as a result of a rising population of immunocompromised patients;
- Antifungal resistant fungal species do not respond to clinical antifungal therapy.

### What this study adds

- The study demonstrates the occurrence of resistant fungal isolates in tuberculosis relapse and retreatment patients that could complicate treatment;
- The current study highlights various fungal etiological agents in pulmonary tuberculosis often missed or misdiagnosed as tuberculosis relapse and often disregarded as tuberculosis culture contaminants.
